# RNF126 in Physiology and Disease: A Multifunctional RING-Type E3 Ubiquitin Ligase in Protein Homeostasis, DNA Repair, and Cancer

**DOI:** 10.3390/cells15131157

**Published:** 2026-06-25

**Authors:** Anh Duc Vu, Shiori Mori, Takeharu Sakamoto

**Affiliations:** 1 Department of Cancer Biology, Institute of Biomedical Science, Kansai Medical University, Osaka 573-1010, Japan; md20221207@kmu.ac.jp (A.D.V.); mori.shr@kmu.ac.jp (S.M.); 2 Faculty of Medical Technology, Hanoi Medical University, Hanoi 100000, Vietnam

**Keywords:** cancer, DNA damage response, male infertility, protein quality control, RNF126, ubiquitin ligase

## Abstract

Ring finger protein 126 (RNF126) is a RING-type E3 ubiquitin ligase that has recently emerged as a multifaceted regulator of cellular homeostasis, stress adaptation, and disease progression. Through its structurally distinct zinc-finger and catalytic RING domains, RNF126 orchestrates substrate recognition and ubiquitin transfer, generating diverse ubiquitin linkages with both proteolytic and nonproteolytic functions. Initially characterized as a component of the protein quality control (PQC) machinery, RNF126 cooperates with chaperones such as BAG6 and UBQLN1 to eliminate mislocalized and misfolded proteins, thereby maintaining proteostasis. Beyond PQC, RNF126 plays pivotal roles in DNA damage response pathways by regulating homologous recombination, non-homologous end joining, checkpoint signaling, and genome stability through substrates, including MRE11, Ku80, RNF168, and 14-3-3σ. Genetic studies have further demonstrated its importance in embryogenesis and male fertility, and accumulating evidence has identified RNF126 as a critical driver of malignancy in multiple cancers. RNF126 promotes tumor progression by degrading or modulating key regulators, such as p21, PTEN, p53, PDKs, and LKB1, thereby enhancing proliferation, metabolic reprogramming, anoikis resistance, metastasis, and chemo/radioresistance. Intriguingly, RNF126 exhibits context-dependent functions, acting as an oncogene or tumor suppressor depending on the tissue type and substrate selection. In addition to cancer, RNF126 has been implicated in neurodegeneration, cardiac pathology, antiviral immunity and adaptive immune regulation. This review summarizes the current knowledge of RNF126 structure, ubiquitin signaling mechanisms, physiological functions, and pathological roles, while discussing emerging therapeutic strategies and future challenges for targeting RNF126 in precision medicine.

## 1. Introduction

Post-translational modification by ubiquitin is a fundamental intracellular mechanism that orchestrates protein degradation, signal transduction, and maintenance of cellular homeostasis [1,2,3]. Protein modification by ubiquitin is catalyzed by a three-enzyme cascade consisting of the E1 ubiquitin-activating enzyme, E2 ubiquitin-conjugating enzyme, and E3 ubiquitin ligase [4]. Within this intricate enzymatic cascade, E3 ubiquitin ligases dictate substrate specificity, acting as pivotal regulators of both normal physiological pathways and pathological processes, including oncogenesis [1,2,5]. Based on their domain structure, E3 ubiquitin ligases are classified into RING (really interesting new gene), HECT (homologous to the E6AP carboxyl terminus), U-box, and RBR (RING-between-RING) types [4]. Among them, RING-type E3 ligases constitute the largest family of ubiquitin ligases, with more than 600 predicted members. RING-type E3 ligases can ubiquitinate not only K48-linked ubiquitin, which promotes proteasomal degradation of the target protein, but also K63-linked or other types of ubiquitin. Among RING-type E3 ligases, ring-finger protein 126 (RNF126) has recently attracted attention because of its diverse pathophysiological roles. With the increasing number of papers published on RNF126 each year and the wide range of biological processes in which RNF126 is involved, it is becoming increasingly difficult to grasp the full picture of RNF126. Thus, in this review, we focus on RNF126, starting with its biochemical properties, followed by its physiological roles, such as protein quality control (PQC), DNA damage response (DDR), and male fertility. Subsequently, we explain the relationship between RNF126 and diseases, especially cancer, and discuss therapeutic strategies targeting RNF126 and future directions of research on RNF126.

## 2. The Molecular Architecture and Ubiquitin Activation of RNF126

**Figure 1:**
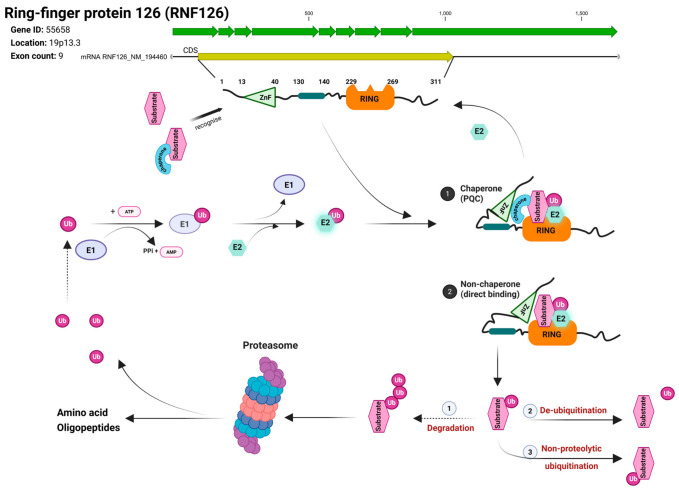
The RING-finger E3 ubiquitin ligase RNF126 orchestrates substrate recognition and ubiquitination via distinct structural domains. The upper panel illustrates the exon organization and coding sequence of RNF126 mRNA, highlighting the N-terminal zinc-finger (ZnF) domain for substrate binding and the C-terminal RING domain for the recruitment of E2 conjugating enzymes. The lower panels depict the canonical ubiquitin cascade, from E1 activation and E2 conjugation to RNF126-mediated ubiquitin transfer to substrates, as well as the dual substrate engagement modes, either through protein quality control (PQC) chaperones or direct binding. RNF126 assembles ubiquitin chains that direct substrates for proteasomal degradation (reversible by deubiquitinating enzymes) or mediate non-proteolytic signaling functions (created in https://BioRender.com accessed on 25 May 2026).

A mechanistic understanding of RNF126-mediated regulation in pathophysiological contexts requires consideration of its structural organization. RNF126 functions as a catalytic enzyme and a multi-domain scaffold that coordinates diverse molecular interactions. The *RNF126* gene is located on chromosome 19p13.3 and consists of nine exons, encoding a 1631-nucleotide mRNA and a 311-amino-acid protein. Although multiple isoforms of RNF126 are annotated, no established functional roles for these isoforms have been reported. This study therefore focused on the canonical 311-amino-acid isoform. Its architecture comprises distinct functional domains that collectively support substrate recognition and catalysis (Figure 1) [6]. The N-terminal zinc finger (ZnF; amino acids 1–40) domain mediates ubiquitin binding and facilitates interactions with target substrates [7,8]. This domain possesses an intrinsic affinity to bind directly to ubiquitin chains and physically engages with chaperones, such as BCL2-associated athanogene 6 (BAG6), allowing RNF126 to recognize and capture specific substrates in a crowded cytosolic environment [8,9]. The central region of the protein (amino acids 101–228) has specific interaction sites that contain an AKT phosphorylation site and mediate direct binding to the protein 14-3-3σ [10,11]. The central region further contributes to its versatility by providing additional interaction interfaces, such as the one used to bind the ubiquilin 1 (UBQN1) chaperone to manage the unimported mitochondrial proteins. The C-terminus has a classic RING finger domain (amino acids 229–269), which adopts a C3H2C3-type structural motif [10] and acts as the catalytic engine [1]. By recruiting specific E2 ubiquitin-conjugating enzymes, the RING domain drives the transfer of ubiquitin molecules onto the captured targets, effectively translating substrate recognition into biochemical action (Figure 1).

RNF126 is characterized by its ability to generate diverse ubiquitin linkages in a context-dependent manner [12]. Depending on the cellular context and the specific E2 enzymes with which it partners, the RING domain can write drastically different biochemical messages [13]. When paired with E2 enzymes, such as UbcH5, it primarily constructs K48 polyubiquitin chains. This ubiquitin linkage serves as a conventional degradation mechanism by the proteasome [13]. Conversely, when RNF126 collaborates with other complexes, such as Ubc13/Uev1a, it ubiquitinates the K63-linked ubiquitin chain on the target protein [13,14]. Interestingly, RNF126 can ubiquitinate rare K27- and K29-linked chains [15]. These non-proteolytic modifications serve as crucial structural scaffolds or activation signals; for instance, they can hyperactivate meiotic recombination 11 (MRE11) nuclease during DNA repair without triggering its degradation [15]. Surprisingly, RNF126 can attach ubiquitin to amino acid residues other than lysine in the target protein. For example, RNF126 ubiquitinates the cysteine, serine, and threonine residues of midnolin (MIDN), resulting in its proteasomal degradation [12]. RNF126 also ubiquitinates cysteine residue 91 of the OTU domain-containing ubiquitin aldehyde-binding protein (OTUB1) [16]. This extraordinary structural flexibility and catalytic ingenuity transform RNF126 from a standard cellular garbage disposal system into a versatile architect of cell survival and disease progression.

## 3. Physiological Indispensability of RNF126

### 3.1. Protein Quality Control (PQC) System and Neurodegenerative Pathology

In healthy cells, RNF126 functions as a key component of the cytosolic PQC network, contributing to the maintenance of cellular homeostasis by preventing the accumulation of mislocalized, misfolded, and aberrant proteins in collaboration with chaperone proteins in the cytosol [8,17]. Central to this process is the recognition of hydrophobic degrons, which are exposed hydrophobic residues that act as recognition signals on aberrant translation products, by chaperone proteins [18,19]. The chaperone protein BAG6 recognizes these hydrophobic degrons and recruits RNF126 via its ubiquitin-like domain at the N-terminus. RNF126 then ubiquitinates BAG6-bound client proteins, committing them to proteasomal degradation [8,17,20,21]. Misfolded endoplasmic reticulum (ER) membrane proteins are extracted from the membrane by valosin-containing protein (VCP)/p97 and then degraded via the BAG6-RNF126 system as part of ER-associated protein degradation (ERAD) [22]. In the case of unimported mitochondrial membrane proteins, another chaperone, UBQN1, recognizes client proteins in the cytosol and recruits RNF126, resulting in the proteasomal degradation of client proteins [23]. These coordinated networks enable RNF126 to efficiently ubiquitinate aberrant proteins with chaperones to maintain proteostasis [8,23,24]. A notable example of this triage mechanism is its involvement in the recognition and clearance of highly toxic, neurodegeneration-associated protein fragments, including those derived from TAR DNA-binding protein of 43 kDa (TDP-43) [19]. These TDP-43 fragments are characterized by exposed hydrophobic residues, necessitating their handling by the BAG6-RNF126 axis to facilitate solubilization and subsequent proteasomal degradation [19]. Additional functions of RNF126 involving BAG6, other chaperones, and non-chaperone proteins are listed in Table 1.

Although it is characterized as a chaperone-dependent E3 ligase in PQC, RNF126 exhibits functional redundancy. Depletion studies have demonstrated that while the BAG6–RNF126 axis significantly contributes to the clearance of misfolded and mislocalized proteins, its loss rarely results in complete substrate stabilization [8]. Despite BAG6–RNF126 depletion, substrate ubiquitination and degradation continue at reduced rates, indicating functional redundancy within the other PQC networks [8]. Alternative pathways, including the CHIP–Hsc70/Hsp90 machinery and the homologous E3 ubiquitin ligase RNF115, can partially compensate for RNF126 loss [7,8,22].

### 3.2. DNA Damage Response (DDR)

Under replication and genotoxic stress, such as irradiation and chemotherapy, cells rely on a coordinated DDR to cope with potentially lethal double-strand breaks (DSBs) [25]. Cells primarily use two major pathways to repair severe DSBs: Homologous recombination (HR) and non-homologous end joining (NHEJ). While HR uses a sister chromatid as a template for error-free repair, NHEJ simply glues the broken ends back together, often resulting in minor mutations. Within the DDR system, the E3 ubiquitin ligase RNF126 functions as an important enhancer of DNA repair, rather than a simple on/off switch [26]. RNF126 promotes HR by associating with the MRE11-RAD50-NBS1 (MRN) complex and ubiquitinates MRE11, thereby increasing its exonuclease activity, generating single-stranded DNA, and activating the ataxia telangiectasia and Rad3-related protein (ATR)-checkpoint kinase 1 (CHK1) signaling cascade [15]. RNF126 has also been reported to cooperate with E2F transcription factor 1 (E2F1) to enhance breast cancer gene 1 (BRCA1) transcription, further supporting HR capacity in irradiated cells [27]. This RNF126-mediated BRCA1 transcription does not require its catalytic activity, and the RNF126 mutant with deletion of amino acid residues 185–195, which are essential for binding to E2F1, functions as a dominant negative. In addition to HR, RNF126 contributes to NHEJ by ubiquitinating Ku80, which promotes the release of the Ku70/80 heterodimer from DSB ends and permits the recruitment of downstream ligation factors required for the completion of NHEJ [28]. Through these activities, RNF126 can accelerate both HR and NHEJ, thereby sustaining DDR signaling and enabling repair in tumor cells exposed to ionizing radiation.

Interestingly, RNF126 also acts as a negative regulator of early DSB signaling. In response to DSBs, RNF126 is recruited to damage sites in an RNF8-dependent manner, where it directly interacts with and ubiquitinates the E3 ligase RNF168 [29]. This modification limits RNF168 stability and activity, thereby reducing the monoubiquitination of histone H2A at lysines 13 and 15 (K13/15), a key signal for the recruitment of p53-binding protein 1 (53BP1) and other mediator proteins to damaged chromatin [29,30]. Consistent with this model, RNF126 overexpression decreased 53BP1, receptor-associated protein 80 (RAP80), and FK2 foci formation after irradiation, whereas RNF126 depletion enhanced RNF168-dependent signaling and 53BP1 accumulation. These findings support a model in which RNF126 restrains the RNF168–53BP1 axis and, by extension, modulates the pathway choice between HR and NHEJ, although the physiological range and tissue specificity of this suppressive activity remain to be fully defined [29,30].

Beyond its enzymatic functions, RNF126 exerts ligase-independent roles in cell cycle control and genome maintenance. After ionizing radiation, RNF126 forms a protein–protein complex with 14-3-3σ via the central region of RNF126, resulting in the mutual stabilization of both proteins and maintenance of the G2/M checkpoint in a p53-independent manner [11]. In this setting, RNF126 protects 14-3-3σ from degradation, while 14-3-3σ limits RNF126 auto-ubiquitination, thereby sustaining checkpoint signaling and providing time for DNA repair before mitotic entry. This function appears to be largely independent of RNF126’s catalytic RING domain, highlighting its scaffolding role in DDR-linked cell cycle control. Recently, RNF126 has been shown to cooperate with another E3 ligase, BRCA1-associated protein (BRAP), to protect genome integrity following DNA damage incurred specifically during late mitosis [31]. In cells irradiated at anaphase/telophase, RNF126 and BRAP undergo ataxia-telangiectasia mutated (ATM)-dependent accumulation and are required for the proper formation of 53BP1 and replication protein A2 (RPA2) foci, resolution of DNA lesions, and long-term survival. These findings suggest that RNF126 functions as a structural scaffold in distinct cell cycle windows, helping cells tolerate DSBs encountered at G2/M and in late mitosis, although the intersection of these roles with its enzymatic activities remains unclear [11,31].

### 3.3. Embryogenesis and Male Fertility

Numerous reports have indicated that RNF126 interacts with various substrates at the cellular level and performs a wide range of functions. However, it is crucial to analyze the role of RNF126 at the organismal level using genetic techniques. The first report on RNF126 knockout mice was published in 2023 [15]. The study demonstrated that RNF126 knockout mice are born at a rate lower than that predicted by Mendelian genetics, indicating that RNF126 plays a critical role in embryonic development; however, the mechanism by which RNF126 contributes to embryogenesis remains unclear.

In addition to embryogenesis, two studies have reported that RNF126 plays an essential role in mammalian spermatogenesis. Liu et al. reported that loss of *Rnf126* impairs meiotic progression and recombination, leading to pachytene arrest and increased apoptosis during spermatogenesis in mice [32]. They also examined whole-exome sequencing in cohorts of patients with nonobstructive azoospermia and oligoasthenoteratozoospermia and identified recurrent RNF126 missense variants (R241H, D253N, and E261A) clustering in exon 8, which encodes the catalytic RING domain essential for E3 ubiquitin ligase activity. All RNF126 variants, except R241H, showed reduced ubiquitination activity, and all three variants were defective in HR activity in vitro. These findings suggest that RNF126-mediated HR activity is essential for spermatogenesis and male fertility. In turn, Wang et al. reported that *Rnf126* depletion results in different types of germ cell reduction, infertility, and microtubule-associated motor activity failure, characterized by spermatozoa with truncated, twisted, and malformed flagella in male mice [33]. Although DNA damage repair was unaltered in *Rnf126*-depleted pachytene spermatocytes, seminiferous tubules of Rnf126 knockout mice showed reduced Bcl2 expression and increased apoptotic cells in their report.

In contrast, phenotypic characterization of female RNF126 knockout mice remains limited. To date, no detailed studies have specifically reported reproductive or developmental defects in female RNF126 knockout mice [32,33]. Whether female RNF126 knockout mice exhibit subtle or context-dependent abnormalities requires further investigation.

Although RNF126 participates in DNA damage repair pathways [15,30], long-term in vivo studies examining cancer susceptibility in RNF126 knockout mice have not yet been reported [32]. Neither aging cohorts nor carcinogen-induced tumor models have been used to assess whether RNF126 deficiency increases the risk of tumorigenesis. Given its roles in genome maintenance, such studies will be important to determine the physiological consequences of RNF126 loss at the organismal level and to clarify whether its deficiency predisposes to cancer development in vivo.

**Table 1:** Substrates and Non-Substrate Interactors of RNF126.

Substrate/Non-Substrate Interactors	Recognition and Recruitment Signal	Cellular Context	RNF126 Expression Effect	Ref
Cytosolic mislocalized proteins and translated aberrant proteins	Chaperone: BAG6	Protein quality control (PQC)	Protein degradation (K48-linked polyUb)	[8,20,21]
Misfolded membrane proteins from the endoplasmic reticulum (ER)	Chaperone: BAG6	PQC	Protein degradation	[22]
Unimported mitochondrial proteins	Chaperone: UBQNL1 (mainly), BAG6 (partly)	PQC	Protein degradation	[23]
Rab8a, Rab10	Chaperone: UBQLN4, BAG6	Primary ciliogenesis, PQC	Protein degradation	[24]
NS3	Chaperone: BAG6	Viral replication	Protein degradation	[34]
G0S2	Chaperone: BAG6	Mitochondrial ATP production during hypoxia	Protein degradation	[35]
FASN	Chaperone: BAG6-GET4	MAPK signaling	Protein degradation	[36]
p21	Direct binding: ZnF and RING domains	Cell cycle	Protein degradation	[9,37,38]
PTEN	Direct binding: RING domain	PI3K/AKT signaling pathway	Protein degradation	[39,40,41]
p53 (wild-type)	Direct binding	proliferation, drug resistance, and cell mobility	Protein degradation	[42]
LKB1	Direct binding	Stemness, migration	Protein degradation	[43]
PDK 1,3,4	Direct binding	Mitochondrial metabolic flux/anoikis resistance	Protein degradation	[44]
ACAP2	unknown	Lipid metabolism	Protein degradation	[45]
MIDN	Direct binding: amino acid 32–229	Tumor malignancy	Protein degradation Non-canonical ubiquitination (C, S, T)	[12]
SLC7A11	Direct binding	Ferroptosis	Protein degradation	[46]
Frataxin	Direct binding: ZnF domain	neurodegeneration	Protein degradation	[47]
IGF-IIR	unknown	cardiac hypertrophy	Protein degradation	[48]
mTOR	Direct binding	proliferation and survival	Protein degradation (K48-linked polyUb)	[49]
**Non-proteolytic ubiquitination (non-degradation) or interaction without ubiquitination**				
AID	Direct binding	unknown	Mono-ubiquitination	[50]
FSP1	Direct binding: amino acids 151–219	plasma membrane localization	Non-proteolytic ubiquitination (K48-linked polyUb)	[51]
MRE11	Direct binding: amino acids 1–100	DNA damage repair (HR)	Non-proteolytic ubiquitination (K27/K29-linked poly Ub)	[15]
RAD50	Direct binding: amino acids 1–100 and 201–311		unknown	
NBS1	Direct binding: amino acids 1–100		unknown	
14-3-3σ	Direct binding: amino acids 130–140	DNA damage response	Stabilization	[11]
E2F1	Direct binding: amino acids 185–195	DNA damage repair (HR)	Enhances E2F1-driven BRCA1 transcription	[27]
MBNL1	Direct binding	Prostate cancer progression/ docetaxel resistance	Regulation of expression levels	[52]
CI-M6PR	Direct binding: ZnF domain	Retrograde endosomal sorting	Regulation of retrograde sorting (K63/K48-linked polyUb)	[53]
ILF3 (i1 isoform)	Direct binding: RING domain	amino acid-mediated mTORC1 signaling.	Non-proteolytic ubiquitination (K63-linked polyUb)	[14]
OTUB1	Direct binding	Antiviral response	Regulation of activity (Non-canonical ubiquitination; C)	[16]
TRAF3	Direct binding: ZnF domain	Antiviral response	Regulation of activity (K63-linked polyUb)	
EGFR	Direct binding: ZnF domain	Endosomal sorting	unknown (K63/K48-linked polyUb)	[13]
Ku70/80	Direct binding	DNA damage repair (NHEJ)	Non-proteolytic ubiquitination (K48-linked polyUb)	[28]
RNF168	Direct binding: ZnF domain	DNA damage response	Non-proteolytic ubiquitination	[30]

## 4. RNF126 and Cancer

### 4.1. A Pan-Cancer Prognostic Biomarker: The Ubiquitous Overexpression and Clinical Significance of RNF126

**Figure 2:**
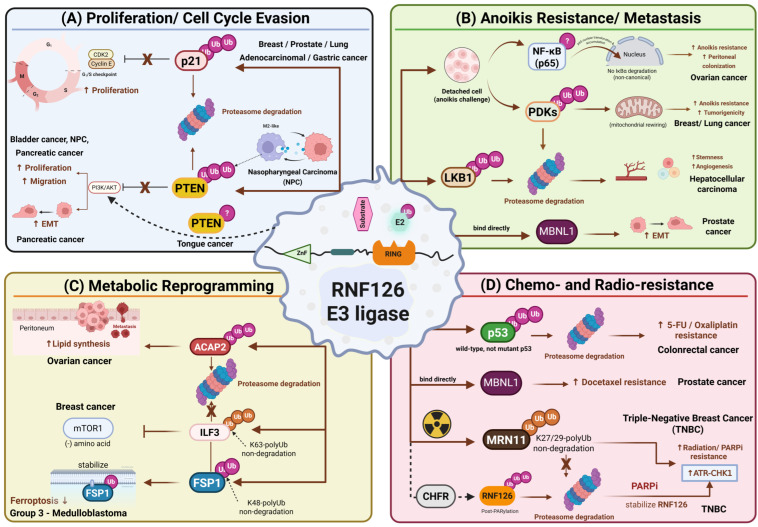
RNF126, as an oncogenic protein, drives multiple cancer hallmarks through ubiquitin-dependent regulation of diverse substrates or direct interaction with non-substrate proteins. It promotes tumor cell proliferation, cell cycle progression, anoikis resistance, metastasis, metabolic reprogramming, and chemo/radio resistance (created in https://BioRender.com accessed on 25 may 2026).

Although *RNF126* is rarely altered at the genomic level through recurrent mutations or copy number changes, its expression is frequently upregulated across a broad spectrum of solid tumors. Earlier studies reported loss of heterozygosity in the 19p13.2–19p13.3 region in breast [54] and ovarian cancers [55]; however, these alterations do not specifically implicate RNF126. Instead, RNF126 dysregulation occurs predominantly through elevated expression at both the mRNA and protein levels in tumor tissues compared with adjacent normal tissues. Clinical and pathological analyses have indicated elevated RNF126 expression in tumor tissues compared with adjacent normal counterparts, including breast [56,57], ovarian [45,58], colorectal [37,42], prostate [52], gastric [38], bladder [39], and lung [44,59] cancers, as well as other cancers [12,40,41,43,51,60]. This pan-cancer upregulation correlates with aggressive clinicopathological features of cancer. In several studies, increased RNF126 expression has been associated with adverse clinicopathological features such as advanced TNM stage, larger tumor size, lymph node metastasis, and vascular invasion. Consistently, multivariate analyses have identified high RNF126 levels as an independent prognostic factor for poor overall survival and disease-free survival in multiple cancer types [38,42,43,52]. Despite this translational promise, RNF126’s prognostic utility remains limited to retrospective cohorts and preclinical evidence from cell line experiments and xenograft tumor models. Large-scale prospective clinical trials are required to validate its diagnostic and prognostic values. In the following sections, we explain how RNF126 is involved in the malignant characteristics of cancer. An overview of this process is illustrated in Figure 2.

### 4.2. RNF126 Is a Therapeutic Vulnerability, Decoding Oncogenic Addiction

**(A)** Proliferation and Cell Cycle Evasion: Cancer cell proliferation frequently relies on RNF126-mediated regulation of key cell cycle and tumor suppressor proteins. RNF126 promotes cell cycle progression, in part, by directly targeting the cyclin-dependent kinase inhibitor p21 for ubiquitin-dependent degradation [9,37,38]. Mechanistically, RNF126 interacts with p21 through its N-terminal (amino acids 1–100) and central (101–200) regions, followed by RING domain–dependent polyubiquitination of p21, leading to its proteasomal degradation [9]. Notably, this process occurs independently of p53 status, as RNF126 retains the ability to degrade p21 in p53-deficient and mutant cancer cells [9]. Consistently, RNF126 depletion stabilizes p21 protein levels without affecting its transcription, resulting in G1 arrest and reduced proliferation and clonogenic survival in breast, prostate, and gastric cancer models [9,38]. In lung adenocarcinoma, RNF126 drives tumor progression via p21 degradation without significantly influencing the invasive potential [59]. Partial rescue of the RNF126 depletion phenotypes upon concomitant p21 depletion further supports p21 as a critical downstream effector of RNF126 [9,37,38,59].

Similarly, RNF126 regulates cell survival pathways through post-translational control of phosphatase and tensin homolog (PTEN) in bladder and pancreatic cancers. RNF126 directly interacts with PTEN via its C-terminal RING-containing region (not the N-terminal) and promotes its ubiquitin-mediated degradation [39]. Loss of PTEN in this context leads to the activation of phosphatidylinositol 3-kinase (PI3K)–AKT signaling and upregulation of G1/S regulators, including cyclin D1. In pancreatic cancer, zinc finger proteins (ZNF) 263 and ZNF31 transactivate RNF126 expression. Increased RNF126 ubiquitination and degradation of PTEN result in AKT-mediated inhibition of glycogen synthase kinase 3β (GSK-3β), stabilization of β-catenin, and activation of proliferative and epithelial–mesenchymal transition (EMT)-associated gene expression [41]. In addition to its intrinsic roles in tumors, RNF126 may also influence the tumor microenvironment. In nasopharyngeal carcinoma, tumor-derived exosomal RNF126 is taken up by tumor-associated macrophages, where it promotes PTEN degradation, activates PI3K–AKT signaling, and drives polarization toward an immunosuppressive M2-like phenotype that further supports tumor growth [40]. RNF126 drives tongue cancer proliferation and progression via the PI3K-AKT pathway. Knockdown of RNF126 decreased AKT1 phosphorylation and nuclear translocation, inhibiting downstream targets, including GSK-3β and forkhead box protein O1 (FOXO1), and significantly reduced tumor burden in vivo. Notably, the specific substrate mediating this effect remains unidentified, with no evidence linking PTEN degradation to this pathway in tongue cancer [60].

Collectively, these findings highlight the context-dependent substrate selectivity of RNF126 and its capacity to coordinately regulate cell-cycle progression, survival signaling, and tumor–immune interactions.

**(B)** Anoikis Resistance and Metastasis: Beyond promoting localized tumor growth, RNF126 has been implicated in multiple processes that facilitate metastatic spread. When cancer cells detach from the extracellular matrix, they must evade anoikis, a form of detachment-induced cell death [61], to survive. In lung and breast cancers, RNF126 has been shown to degrade pyruvate dehydrogenase kinases (PDKs), thereby reprogramming energy metabolism between glycolysis and mitochondrial oxidative phosphorylation to support survival under anchorage-independent conditions [44]. RNF126 also contributes to epithelial–mesenchymal transition (EMT) and the maintenance of cancer stem cell–like properties. In prostate cancer, RNF126 decreases the transcription of muscleblind-like splicing regulator 1 (MBNL1), thereby promoting EMT [52]. RNF126 can also bind to MBNL1; however, the function of this binding remains unclear. In hepatocellular carcinoma, it targets the tumor suppressor liver kinase B1 (LKB1) for degradation, thereby sustaining cancer stem cell-like properties and promoting angiogenesis [43]. In our previous study on ovarian cancer, RNF126 was shown to strongly activate the NF-κB signaling pathway under non-adherent conditions that mimic detachment from the extracellular matrix (ECM). This activation promoted anoikis resistance and facilitated aggressive peritoneal colonization. Interestingly, in these ovarian cancer models, RNF126-mediated NF-κB activation is associated with nuclear accumulation of p65 in the absence of canonical inhibitor of nuclear factor kappa B alpha (IκBα) degradation [58]. This observation suggests the involvement of non-canonical or IκBα-independent mechanisms of NF-κB activation, although the precise molecular events remain to be elucidated. Whether RNF126 directly ubiquitinates p65 to modulate its subcellular localization or acts on upstream regulatory factors remains an open question that warrants further investigation. Clarifying these mechanisms may offer insights into therapeutic strategies for targeting peritoneal metastasis in ovarian and other abdominal cancers.

**(C)** Metabolic Reprogramming: RNF126 regulates lipid metabolism by ubiquitination and degradation of ACAP2 (ArfGAP with coiled-coil, ankyrin repeat, and PH domains 2) in ovarian cancer. Co-immunoprecipitation studies confirmed a direct interaction between RNF126 and ACAP2, leading to ACAP2 proteasomal degradation. Loss of ACAP2 results in increased intracellular lipid accumulation, elevated triglyceride and cholesterol levels, and upregulation of lipogenic enzymes, including fatty acid synthase, acetyl-CoA carboxylase, and stearoyl-CoA desaturase [45]. In murine xenograft and metastasis models of ovarian cancer, RNF126 depletion significantly reduced tumor growth and lung metastatic burden. Notably, concomitant ACAP2 silencing largely rescued these phenotypes, indicating that ACAP2 is a key downstream effector of RNF126. However, the molecular mechanisms linking ACAP2 loss to the transcriptional activation of lipogenic programs remain unclear [45].

In Group 3 medulloblastoma, a particularly aggressive pediatric brain tumor, RNF126 contributes to ferroptosis resistance by regulating the subcellular localization of ferroptosis suppressor protein 1 (FSP1) [51]. Through direct interaction with FSP1, RNF126 promotes its retention at the plasma membrane, preventing lipid peroxidation and enabling tumor cells to evade ferroptosis. Notably, RNF126 mediates spatial regulation by conjugating K48-linked polyubiquitin chains to FSP1. Although K48-linked ubiquitination is classically associated with proteasomal degradation, this modification of FSP1 appears to be non-proteolytic and functions as a membrane-anchoring signal [51].

In MCF7 breast cancer cells, RNF126 regulates nutrient sensing and cell growth through the interleukin enhancer-binding factor 3 (ILF3)–mechanistic target of rapamycin complex 1 (mTORC1) pathway. Under the amino acid-depleted condition, RNF126 catalyzes K63-linked polyubiquitination of ILF3, enhancing its interaction with the GAP activity toward Rags 2 (GATOR2) complex and resulting in mTORC1 suppression [14]. Functionally, RNF126 depletion in 3D spheroid cultures impaired MCF7 cell proliferation and migration, with enhanced sensitivity to the mTORC1 inhibitor rapamycin. This negative regulation of mTORC1 by an oncogenic E3 ligase suggests that RNF126 may serve as an adaptive metabolic brake under nutrient-limited conditions, preventing metabolic collapse and promoting tumor cell survival during periods of stress. However, the precise conditions that activate this regulatory axis and its role in therapy resistance remain to be elucidated. Although the experiments were performed in 3D spheroid cultures, which can involve anchorage-independent growth, the main findings focus on defects in amino acid sensing and mTORC1-dependent proliferation rather than anoikis resistance. The cited study did not directly examine anoikis, so there is no clear connection to the anoikis data discussed elsewhere in the manuscript [14].

Further investigation is required to determine the conditions under which this regulatory axis is engaged and its contribution to therapeutic resistance.

**(D)** Chemo- and Radioresistance: RNF126 plays a significant role in mediating therapeutic resistance across multiple cancer contexts. In prostate cancer, the RNF126–MBNL1 axis enhances resistance to docetaxel [52]. Similarly, in colorectal cancer (CRC), RNF126 promotes the degradation of wild-type p53, thereby contributing to reduced sensitivity to 5-fluorouracil and oxaliplatin [42]. Furthermore, in triple-negative breast cancer (TNBC), RNF126 interacts with the MRN complex and ubiquitinates MRE11, which activates the ATR-CHK1 signaling pathway and enhances resistance to ionizing radiation [15] and poly (ADP-ribose) polymerase (PARP) inhibitors [3]. However, several aspects of RNF126 biology remain unclear. For example, although RNF126 mediates the degradation of wild-type p53 in CRC, it appears to exhibit limited activity against mutant p53 variants [42]. However, the mechanistic basis for this apparent selectivity remains unclear. In addition, while RNF126 promotes HR and may therefore reduce the efficacy of PARP inhibitors (PARPi), recent evidence suggests that RNF126 itself can be targeted for degradation by the checkpoint with forkhead and RING finger domains (CHFR) protein following PARP1-mediated PARylation [3]. This observation suggests the existence of a regulatory feedback mechanism that may complicate therapeutic targeting strategies. Taken together, these findings highlight the need for further investigation into the context-dependent functions of RNF126, particularly its substrate specificity and interaction with PARP1-related pathways, to better assess its potential as a target for enhancing chemo- and radiosensitivity.

### 4.3. Context-Dependent RNF126 Function

RNF126 exhibits a striking context-dependent functional duality in various malignancies. In epithelial cancers, including breast, prostate, colorectal, and bladder tumors, RNF126 predominantly acts as an oncogenic E3 ligase. Its pro-tumorigenic role is driven by the ubiquitin-mediated degradation of key tumor suppressors, such as p21, p53, and PTEN, thereby promoting cell cycle progression, chemoresistance, and activation of the PI3K/AKT/mTOR axis. Consistently, RNF126 overexpression in these settings correlates with aggressive disease and poor clinical outcomes in various types of cancer [15,37,38,40,41,42,43,51,56,57,59,62]. In CRC, RNF126 behaves as a p53-conformation-selective E3 ligase that preferentially targets wild-type p53, but not its mutant counterparts, with a direct impact on p21-mediated cell-cycle control and chemoresistance. In p53 wild-type CRC cells, RNF126 promotes ubiquitination and proteasomal degradation of p53, leading to reduced p21 transcription, loss of the G1/S checkpoint, and increased resistance to DNA-damaging agents such as 5-fluorouracil and oxaliplatin. In contrast, in mutant p53-harboring CRC lines (e.g., Colo-205 and SW620), RNF126 overexpression did not alter p53 or p21 levels, indicating that its E3 activity toward p53 is largely confined to the wild-type conformation and that mutant p53 effectively escapes this regulatory layer. This striking selectivity illustrates the tissue-specific context in which RNF126 can efficiently eliminate wild-type p53 in the colorectal epithelium while leaving mutant p53 intact. However, the structural and microenvironmental determinants of this discrimination remain poorly defined, limiting rational p53-stratified targeting strategies in CRC [42].

In contrast, in germ-cell and hematological malignancies, RNF126 exerts tumor-suppressive functions. In testicular germ cell tumors, RNF126 facilitates MIDN degradation via non-canonical ubiquitination. MIDN exerts proteasomal degradation of target proteins, such as the transcriptional factor early growth response protein 1 (EGR1), by bypassing the ubiquitin-mediated system [63]. RNF126-mediated MIDN degradation leads to EGR1 accumulation and subsequent restoration of p53 and PTEN expression [12]. Similarly, in acute and chronic myeloid leukemias, RNF126 targets mTOR for K48-linked ubiquitination and proteasomal degradation, thereby impairing leukemic cell survival by disrupting metabolic and autophagic homeostasis, underscoring its antitumor role in this context [49].

This functional divergence highlights a fundamental unresolved question: how does RNF126 selectively target distinct substrates to either activate or suppress the same signaling axis in a tissue-specific manner? The opposing regulation of the PTEN-mTOR pathway, via PTEN degradation in epithelial cancers versus direct mTOR degradation in leukemia, illustrates a critical gap in our understanding of RNF126 substrate selection and its regulatory mechanisms. From a translational perspective, this context dependency has important therapeutic implications. Global inhibition of RNF126 is unlikely to be universally beneficial and may have deleterious effects on various tissues. Instead, therapeutic strategies should be tailored to tumor context-targeting RNF126 activity in epithelial cancers while preserving or restoring its function in hematological and germ cell malignancies.

## 5. RNF126 and Other Diseases

### 5.1. Friedreich Ataxia

Friedreich ataxia (FRDA) is a devastating neurodegenerative disease caused by a critical shortage of the mitochondrial protein, frataxin [64]. In this context, RNF126 acts as a ruthless executioner by directly ubiquitinating and destroying the remaining frataxin precursors [47]. Strikingly, silencing RNF126 successfully restored frataxin protein levels in FRDA patient-derived cells. This implies that RNF126 may be a therapeutic target for FRDA, although no further functional analysis was conducted in this study.

### 5.2. Cardiac Hypertrophy and Myocardial Ischemia

In addition, RNF126 has been reported to protect against cardiac hypertrophy by promoting the degradation of insulin-like growth factor II receptor (IGF-IIR), which functions as a scavenger receptor that mediates the clearance of IGF-II [48]. However, angiotensin II (ANG II) activates the extracellular signal-regulated kinase (ERK)/GSK3 signaling pathway, leading to the phosphorylation of heat shock factor 1 (HSF1). This phosphorylation promotes the degradation of RNF126, resulting in the accumulation of IGF-IIR on the cell surface and subsequent cardiac hypertrophy and apoptosis. However, the mechanism by which HSF1 selectively drives RNF126 degradation remains entirely unresolved in this study. From a clinical perspective, this suggests a clear therapeutic implication: rather than targeting RNF126 directly, interventions should focus upstream, such as inhibiting GSK3 or blocking ANG II type I receptor signaling to prevent aberrant HSF1 phosphorylation and thereby preserve RNF126 function.

During myocardial ischemia and severe hypoxia, cardiomyocytes critically depend on G0/G1 switch gene 2 (G0S2) to sustain mitochondrial ATP production and survive energy stress [65]. However, the RNF126–BAG6 quality control machinery actively targets G0S2 for degradation [35]. Silencing RNF126 increased G0S2 protein levels, which retained mitochondrial ATP production in cardiomyocytes exposed to hypoxia. BAG6 silencing also increased G0S2 protein levels but failed to retain mitochondrial ATP production during hypoxia. This implies that BAG6 is also necessary for G0S2 function during hypoxia and that targeting RNF126 may be more effective for preventing myocardial ischemia than BAG6 inhibition.

### 5.3. Immune Responses

Beyond cancer, RNF126 regulates antiviral responses and B-cell antibody maturation. RNF126 promotes tumor necrosis factor receptor-associated factor 3 (TRAF3) K63 ubiquitination to activate antiviral signaling while contributing to ERAD-mediated quality control that influences immune protein homeostasis. In flavivirus (Zika/Dengue virus) infections, RNF126 participates in host ERAD responses that restrict viral replication. RNF126 ubiquitinates viral nonstructural protein 3 (NS3) via K48-linked polyubiquitination in association with BAG6. This impairs NS3 protease activity, resulting in decreased Zika/Dengue virus replication [34]. TRAF3 is a key adaptor in type I interferon signaling, which is crucial for controlling viral infections [66]. RNF126 binds to TRAF3 and promotes K63-linked polyubiquitination [16]. Interestingly, RNF126 also ubiquitinates OTUB1 at cysteine residue 91, which is essential for deubiquitinase activity. This prevents OTUB1 from deubiquitinating K63 polyubiquitination of TRAF3, which is necessary for type-I interferon production. Thus, RNF126 may also contribute to antiviral immunity through the OTUB1-TRAF3 axis. Regarding adaptive immunity, RNF126 has been reported to ubiquitinate activation-induced cytidine deaminase (AID), an essential enzyme for antibody diversification in B cells [50]. The functional importance of this RNF126-mediated ubiquitination of AID has not been further addressed; thus, whether RNF126 affects antibody diversity remains unclear.

## 6. Translating RNF126 into Precision Theranostics

### 6.1. Diagnostic Value and the Structural Bottleneck

As a powerful biomarker, high RNF126 expression predicts poor prognosis in many types of cancer [15,37,38,40,41,42,43,51,56,57,59,62]. However, pharmacologically targeting RNF126 presents a formidable structural challenge, as its catalytic RING domain is characteristically flat and lacks deep hydrophobic pockets amenable to conventional small-molecule inhibition [1,67]. Exacerbating this challenge, RNF126 and RNF115 share 75% identity in their RING domains [2]. This redundancy poses a major obstacle to the development of specific inhibitors of RN126 enzymatic activity.

### 6.2. Indirect Therapeutic Strategies

**Figure 3:**
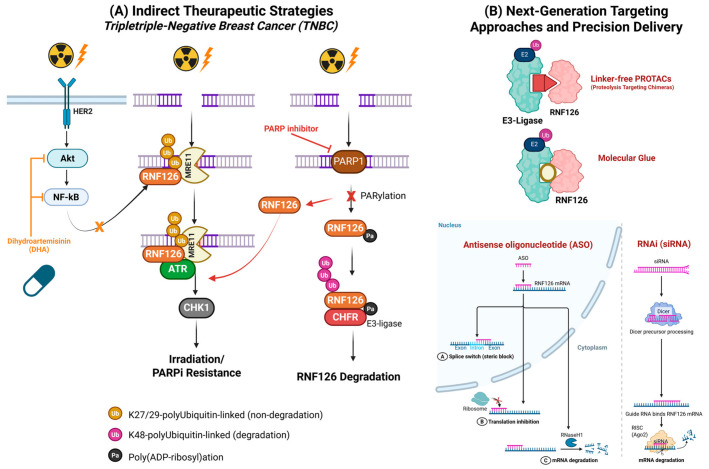
Indirect therapeutic interventions, as illustrated for triple-negative breast cancer (TNBC), exploit the upstream and downstream signaling mechanisms of RNF126. Precise targeting with dihydroartemisinin (DHA) can enhance the efficacy of radiotherapy, while direct inhibition of RNF126 (e.g., by siRNA) resensitizes TNBC cells to PARP inhibitors and irradiation. Next-generation strategies include the development of RNF126-specific linker-free PROTACs or molecular glues to selectively degrade or modulate RNF126, as well as the use of antisense oligonucleotides (ASOs) and RNAi to inhibit RNF126, thereby stabilizing and protecting tumor suppressor proteins. (Created in https://BioRender.com accessed on 25 May 2026).

Given the challenges associated with direct inhibition of RNF126, alternative strategies that target its functional dependencies or upstream regulators have been proposed. One potential approach is based on synthetic lethality. Tumors with RNF126 overexpression often exhibit elevated replication stress, which may increase their reliance on the ATR-CHK1 pathway for survival (Figure 3A). Inhibiting CHK1 selectively triggers genetic collapse, killing aggressive cells [57]. Another strategy is to increase tumor sensitivity to DNA damage. Since loss of RNF126 impairs homologous recombination, tumors with low RNF126 expression may respond better to radiation and PARP inhibitors [3,15]. However, there is feedback from PARPi to the RNF126. PARP1 normally attaches poly(ADP-ribose) chains to RNF126, recruiting CHFR ligase to destroy it. Consequently, administering a PARPi inadvertently blocks this degradation, stabilizing and upregulating RNF126, which may grant the tumor a survival advantage and drive resistance [3]. Alternatively, blocking the signaling pathways that drive RNF126 expression may also be beneficial. In triple-negative breast cancer, RNF126 is induced by radiation through the HER2-AKT-NF-κB pathway [15]. Dihydroartemisinin, which inhibits both AKT and NF-κB, has been reported to reduce RNF126 levels and improve the response to radiation (Figure 3A). The ERK-ELK1 signaling pathway also boosts RNF126 transcription in breast and lung cancers [44]. Thus, inhibitors of this pathway may be useful in these types of cancers. Nevertheless, these therapeutic strategies are still largely based on in vitro findings. More comprehensive preclinical studies, including genetically engineered mouse models and well-designed xenograft experiments, will be needed to better understand the role of RNF126 in different cancer contexts before considering it as a therapeutic target.

### 6.3. Next-Generation Targeting Approaches and Precision Delivery

Targeted protein degradation has emerged as a promising strategy to modulate RNF126 activity (Figure 3B). Recent studies have explored the use of molecular glues containing covalent fumarate handles to recruit RNF126 for the degradation of otherwise undruggable oncoproteins [68]. However, this approach faces significant challenges. Because of the high conservation of zinc-finger domains among E3 ligases, these covalent molecules may also engage other structurally related ligases, raising concerns about off-target protein degradation and unintended disruption of the ubiquitin system [67,68]. To address the large size of conventional proteolysis-targeting chimeras (PROTACs), researchers have developed smaller, linker-free PROTACs that utilize single-amino-acid degradation signals. These compact molecules significantly enhance target-ligase interactions and oral bioavailability [69]. Another approach is to target RNF126 expression using therapeutic oligonucleotides, such as antisense oligonucleotides and siRNA [70,71] (Figure 3B). Although therapeutic oligonucleotides against RNF126 have not yet been reported, they are applicable to genes whose proteins have high similarity with other family proteins. Nevertheless, a significant translational gap remains in terms of systemic safety. As RNF126 is indispensable for male fertility and protein quality control, systemic inhibition could trigger severe adverse effects. Therefore, any therapeutic strategy would likely require tumor-selective delivery systems, such as antibody–drug conjugates or lipid nanoparticles, to minimize toxicity to normal tissues. Moreover, most current evidence is based on in vitro studies. Additional functional investigations (e.g., using CRISPR-based tools to precisely modulate RNF126 in different cellular and physiological contexts) will be necessary to better define its roles before targeted degradation strategies can be meaningfully pursued.

## 7. Future Perspectives and Conclusions

RNF126 has emerged as an important regulator with diverse functions across different cellular contexts. Through its versatile ubiquitin ligase activity, RNF126 can aggressively promote cancer progression and therapy resistance while simultaneously protecting neurons from toxic protein aggregates and supporting normal spermatogenesis. Numerous studies have been conducted on the function of RNF126. However, the following points need to be addressed in future studies to better understand and target RNF126 for disease control.

First, comprehensive proteomic analyses to identify the substrates and binding proteins of RNF126 are essential to understand the biological functions of RNF126 and its regulatory mechanisms. In addition to classical immunoprecipitation approaches, in situ proximity-dependent labeling techniques, such as E3-substrate tagging by ubiquitin biotinylation (E-STUB) and proximity- and orientation-dependent tagging of Ub (Ub-POD), are useful for identifying weak and transient binding partners/substrates of RNF126 [72,73,74]. Ubiquitin-activated interaction traps (UBAITs), which express RNF126 fused to ubiquitin at the C-terminus and thereby covalently trap their substrates in a stable complex, may also be effective [75]. Because RNF126 is involved in PQC and cancer malignancy, it is intriguing to compare the substrates/binding proteins of RNF126 in the same cells under normal and stress conditions to understand the situation-dependent roles of RNF126.

Second, the mechanisms governing RNF126 expression need to be elucidated. RNF126 is overexpressed in many cancers and is highly expressed in the testis among normal organs. While it is known that its expression is promoted in cancer by ERK signaling and HER2/AKT/NF-κB signaling activated by irradiation [15,44], the cell-type-specific mechanisms underlying its expression remain unclear. In particular, the role of epigenetic regulation in controlling RNF126 expression has not been well characterized. Clarifying these mechanisms would help us better understand how RNF126 expression is turned on or off in different tissues and disease states, which may ultimately provide insights into its biological functions and potential as a therapeutic target.

Third, elucidating the role of RNF126 at the organismal level remains an important but underexplored area. Although most functional studies on RNF126 have been performed in cultured cells, its physiological roles in living organisms are still not well defined. Germline RNF126 knockout mice exhibit defects in embryonic development and male fertility; however, they do not display major outward abnormalities [15,32,33]. While numerous studies have shown that RNF126 is crucial for protein quality control mediated by chaperones such as BAG6 and UBQN1 at the cellular level, the phenotypic abnormalities observed at the organismal level are relatively mild. Although these mice exhibit defects in embryonic development and male fertility, they do not display major abnormalities in most other tissues. This discrepancy suggests that other proteins or molecular pathways may compensate for the loss of RNF126 under normal physiological conditions. Since germline RNF126 knockout mice are obtained at a lower-than-expected Mendelian ratio [15], making analysis difficult. To better uncover the critical functions of RNF126, it would be valuable to study germline or conditional knockout models under various stress conditions, such as proteotoxic stress, oxidative stress, or DNA damage. In addition, the use of cell-type-specific or inducible conditional knockout mice would be valuable for investigating the role of RNF126 in disease models such as cancer, viral infection, cardiovascular disease, and neurological disorders. Furthermore, because zebrafish Rnf126 is highly conserved, showing 62% sequence identity with human RNF126 [50], this model may also provide a useful platform for studying the in vivo functions of Rnf126 at the organismal level.

In conclusion, RNF126 is a versatile ubiquitin E3 ligase that is physiologically involved in PQC, DDR, and male fertility. In turn, RNF126 overexpression promotes malignancy in many types of cancer. Thus, a better understanding of the pathophysiological roles of RNF126 and its regulatory mechanisms would be helpful in developing further clinical applications targeting RNF126.

## Data Availability

No new data were created or analyzed in this study.

## Abbreviations

The following abbreviations are used in this manuscript.

53BP1	p53-binding protein 1
ACAP2	ArfGAP with coiled-coil, ankyrin repeat, and PH domains 2
ANG II	angiotensin II
AID	activation-induced cytidine deaminase
ATM	ataxia-telangiectasia mutated
ATR	ataxia telangiectasia and Rad3-related protein
ASO	antisense oligonucleotide
BAG6	BCL2-associated athanogene 6
BRAP	BRCA1-associated protein
BRCA1	breast cancer gene 1
CHFR	checkpoint with forkhead and RING finger domains
CHK1	checkpoint kinase 1
CRC	colorectal cancer
DDR	DNA damage response
DSB	double-strand break
E2F1	E2F transcription factor 1
EGR1	early growth response protein 1
EMT	epithelial–mesenchymal transition
ER	endoplasmic reticulum
ERAD	endoplasmic reticulum-associated protein degradation
ERK	extracellular signal-regulated kinases
E-STUB	E3-substrate tagging by ubiquitin biotinylation
FOXO1	forkhead box protein O1
FRDA	Friedreich ataxia
FSP1	ferroptosis suppressor protein 1
G0S2	G0/G1 switch gene 2
GATOR2	GAP activity toward Rags 2
GSK-3β	glycogen synthase kinase-3β
HECT	homologous to the E6AP carboxyl terminus
HR	homologous recombination
HSF1	heat shock factor 1
IGF-IIR	insulin-like growth factor II receptor
IκBα	inhibitor of nuclear factor kappa B alpha
ILF3	interleukin enhancer-binding factor 3
LKB1	liver kinase B1
MIDN	midnolin
MRE11	meiotic recombination 11
MRN	MRE11-RAD50-NBS1
mTOR	mechanistic target of rapamycin
mTORC1	mechanistic target of rapamycin complex 1
NF-κB	nuclear factor kappa-light-chain-enhancer of activated B cells
NHEJ	non-homologous end joining
NS3	nonstructural protein 3
OTUB1	OTU domain-containing ubiquitin aldehyde-binding protein
PARP	poly (ADP-ribose) polymerase
PARPi	poly (ADP-ribose) polymerase inhibitor
PDK	pyruvate dehydrogenase kinase
PI3K	phosphatidylinositol 3-kinase
PQC	protein quality control
PROTAC	proteolysis targeting chimera
PTEN	phosphatase and tensin homolog
RAP80	receptor-associated protein 80
RING	really interesting new gene
RBR	RING-between-RING
RNAi	RNA interference
RNF126	Ring-finger protein 126
RPA2	replication protein A2
TDP-43	TAR DNA-binding protein of 43 kDa
TNBC	triple-negative breast cancer
TRAF3	tumor necrosis factor receptor-associated factor 3
UBAIT	ubiquitin activated interaction trap
Ub-POD	proximity-and orientation-dependent tagging of Ub
UBQN1	ubiquilin 1
VCP	valosin-containing protein
ZnF	zinc finger
ZNF	zinc finger protein
